# Associations between refractive error components and higher-order aberrations in simple myopia and compound myopic astigmatism

**DOI:** 10.3389/fopht.2025.1532931

**Published:** 2025-03-20

**Authors:** Sahar Mohaghegh, Shahram Bamdad, Haleh Kangari, Saeed Rahmani

**Affiliations:** ^1^ Department of Optometry, School of Rehabilitation, Shahid Beheshti University of Medical Sciences, Tehran, Iran; ^2^ Department of Ophthalmology, Poostchi Ophthalmology Research Center, School of Medicine, Shiraz University of Medical Sciences, Shiraz, Iran

**Keywords:** higher-order aberration, myopia, astigmatism, coma, spherical aberration

## Abstract

**Aim:**

To investigate associations between refractive error components and higher-order aberrations (HOAs) in adult myopic subjects.

**Methods:**

A total of 1370 myopia right eyes, aged 18-40, were included in a cross-sectional study. Subjective cycloplegic refractions and distance aberrometry measured with a Shack-Hartmann device were analyzed. Zernike components of horizontal coma (Z_3_^1^), vertical coma (Z_3_^-1^), oblique trefoil (Z_3_^3^), vertical trefoil (Z_3_^-3^), spherical aberration, and total root-mean-square (RMS) wave-front error for 6 mm pupil were analyzed. Pearson’s correlations were calculated between sphero-cylindrical components and HOAs based on vector analysis for the astigmatism axis. Total subjects were divided into two subgroups: simple myopia (SMY, 648 eyes) and compound myopic astigmatism (CMA, 722 eyes). HOAs were compared between the two subgroups.

**Results:**

Total RMS wave-front error correlates with spherical equivalent myopia (r = -0.1, P<0.05) and J45 (r = 0.1, P<0.001). J0 correlates positively with vertical coma (Z_3_^-1^) (r = 0.1 p <0.001) and negatively with oblique trefoil (Z_3_^3^) and vertical trefoil (Z_3_^-3^), (r = -0.1, p < 0.001; r = -0.1, P < 0.05). The total RMS wavefront-error was larger in the CMA (|0.37| ± 0.18 µm) compared to the SMY (|0.34| ± 0.16 µm, P <0.001). The mean values of vertical coma (Z_3_^-1^), vertical trefoil (Z_3_^-3^), and oblique trefoil (Z_3_^3^) differed between the two subgroups.

**Conclusion:**

Total RMS wave-front error increases with increasing myopia and astigmatism. Increasing myopia power does not show a systematic correlation with HOAs components. A weak systematic correlation is suggested between astigmatism direction and third-order aberrations.

## Introduction

Degraded retinal image quality (1) and hyperopic defocus (2, 3) have been shown to trigger axial length elongation in numerous animal models. In humans, image degradation due to low-order aberrations such as hyperopia, myopia, and astigmatism can be corrected by sphero-cylindrical lenses. However, degraded retinal image quality due to higher-order aberrations (HOAs) remains unsolved, at least with spectacles (4). Previous studies have suggested that HOAs may provide optical signals that contribute to developing sphero-cylindrical refractive error (5, 6). For example, it was suggested that increasing negative spherical aberration (Z_4_^0^) with accommodation might significantly reduce the retinal image quality and promote near-work-induced myopia (7). Also, it has been hypothesized that the combination of negative-spherical aberration (Z_4_^0^), negative-vertical coma (Z_3_^-1^), and positive-oblique trefoil (Z_3_^3^) may signal the development of myopia and with-the-rule (WTR) astigmatism (8). Therefore studying the relationship between HOAs and refractive errors has significance.

The literature on HOAs and their relationship to refractive errors is ambivalent. Some studies do not support an association between HOAs and refractive errors (9–11). For example, it was reported that evidence for higher levels of monochromatic aberrations in myopes in comparison with other refractive subgroups is weak (10), and root mean square (RMS) wave-front errors of various HOAs in myopic and hyperopic eyes are uncorrelated with refractive error (9, 11) or cross-sectional studies do not support evidence of relationships between emmetropization and ocular aberrations (12). On the other hand, some studies support an association between HOAs, myopia, and astigmatism. For example, It was found that twenty percent of myopic adults had total RMS wave-front error values greater than values for all of the emmetropic adults (13). Also, it was reported that higher levels of myopia tend to have a smaller value of spherical aberration (Z_4_^0^) (14). In a previous study on subjects with well-established refractive errors, it was indicated that corneal HOAs do not correlate with myopia, emmetropia, and hyperopia, but it has a meaningful correlation with astigmatism (15).

If there were a link between low-order and HOAs, an association between these interactions and the degree of refractive error would be expected. Maybe the complex interactions between HOAs and low-order aberration for the different refractive error types do not allow for uncovering potential associations if all types of myopic refractive errors will be analyzed in one group. Therefore, in this study, we first aim to find the correlation between HOAs and sphero-cylindrical components. Then we aim to compare HOAs between simple myopia (SMY) and compound myopic astigmatism (CMA) subgroups.

## Materials and methods

A cross-sectional study design was used to investigate myopic subjects aged 18-40 years old in a total of 1370 right eyes, which were scheduled for photorefractive keratectomy surgery at the cornea clinic in the Shiraz University of Medical Science from 2018-2020. The study was approved by the Shahid Beheshti University of Medical Sciences ethics committee (code: IR.SBMU.RETECH.REC.1401.055) and was under the tenet of the declaration of Helsinki. Informed consent was obtained from all the participants.

Patients who used soft contact lenses were instructed to discontinue wearing them at least one month before the measurements. For the few cases that might have used orthokeratology lenses, they were also asked to discontinue the use of orthokeratology lenses at least two months before the measurements. Patients with severe dry eye were not considered suitable candidates for PRK and were excluded from the study. Patients with moderate to mild dry eye were first treated for the condition, and measurements were taken after the treatment, when the patients were prepared to undergo PRK.

Two drops of cyclopentolate 1%, five minutes apart applied for cycloplegic refraction. Cycloplegic refraction was performed 20 minutes after the second drop. Auto refraction was performed with Nidek ARK-1 auto refractometer (Nidek Technologies, Gamagori, Japan). After performing auto refraction, the manual retinoscopy with retinoscope (Hine Beta 200, Germany) was performed for all participants. The measurement of subjective refraction after applying cycloplegia was taken for analysis. Comprehensive ocular examination was performed for all candidates, including dilated fundus examination, slit-lamp bio-microscopy, and applanation tonometry. The binocularity state was checked with the cover test. Corneal imaging with the Pentacam-HR (Oculus Optik- geräte GmbH, Wetzlar, Germany) was performed for all candidates to evaluate for ectatic corneal diseases. Patients with ectatic corneal disease, strabismus, and any ocular conditions affecting visual acuity were not registered as candidates for refractive surgery and were excluded from this analysis.

Aberrometry for 6 mm pupil diameter was performed with a commercially available Hartmann-Shack wavefront aberrometer (Bausch & Lomb Zywave, Rochester, NY) for all subjects. Measurements were done after the instillation of two drops of cyclopentolate 1% five minutes apart to control the accommodation response, and patients were asked to perform a full blink before measurements. Total RMS wave-front error up to Zernike’s 4th order was captured. Values for Zernike coefficients of third-order horizontal & vertical coma (Z_3_^1^, Z_3_^-1^), third-order oblique & vertical trefoil (Z_3_^3^, Z_3_^-3^), and fourth-order primary spherical aberration (Z_4_^0^) were available for analysis. Zernike coefficients were multiplied by -1 and transformed into the standard form recommended by the optical society of America (16).

Data from patients registered as refractive surgery candidates were used as the study population. Inclusion criteria were SMY and CMA of individuals aged 18-40. Exclusion criteria were simple astigmatism, hyperopia, and hyperopic astigmatism individuals due to their small sample size. Since aging can affect HOAs in patients older than 40 due to crystalline lens changes, they were also not included in the study. Due to the mirror symmetry between the right and left eyes’ ocular aberrations (17), only right-eye data were selected for analysis.

SMY was defined as myopia power ≤ -0.50 D and astigmatism power ≥ |0.50| D. CMA was defined as myopia power ≤ -0.50 D and astigmatism power < -0.50 D. The magnitude of HOAs was compared between SMY and CMA subgroups. To consider the astigmatism axis, the astigmatism components were calculated as J0 and J45 with the formula of J0 = (-cylinder/2) ρ (cos2A) and J45 = (−cylinder/2) ρ (sin2A), while A= astigmatism axis. Positive values of J0 indicate WTR astigmatism, and negative values of J0 indicate against-the-rule (ATR) astigmatism. Spearman’s correlations between refractive error components (sphere, J0, and J45) and HOAs were evaluated. Multiple linear regression was applied to investigate the effect of HOAs on spherocylindrical components controlling for age, gender, and corneal characteristics including mean front and back corneal curvatures, central corneal thickness, white-to-white corneal diameter, and anterior chamber depth. Then, the total study subjects were divided into two subgroups: SMY and CMA. HOAs were compared between the two subgroups.

### Statistical analysis

Statistical analysis was performed using SPSS software version 18.2.2. (Chicago Inc., IL, USA). Descriptive statistics were applied to describe features of the studied population. The Shapiro-Wilk test was used to evaluate the normality of data. Spearman’s correlation test was applied to find correlations between sphero-cylindrical components and HOAs. Bonferroni correction was used for several correlations, and calculated P-values were multiplied by 18 due to applying 18 correlations. Corrected P-values are provided. P < 0.05 is considered significant. Multiple linear regression analysis was applied. The Mann-Withney U test was used to compare HOAs between SMY and CMA subgroups. P < 0.05 is considered significant.

## Results

**Table 1** shows the total and within groups’ demographic data. The mean age ± standard deviation (SD) was 29.01 ± 5.19. 648 eyes were in the SMY subgroup, and 722 eyes were in the CMA subgroup. The mean spherical equivalent (SEQ) ± SD was -3.64 ± 1.59 D, range [-0.75 D to -10.00 D] in the total population. The mean cylindrical power was -1.01 ± 0.95 D, range [0 D to -5.00D]. The mean SEQ ± SD were -3.31 ± 1.37 D and -4.07 ± 1.64 in SMY and CMA, respectively. The mean ± SD of age, sphere, and cylindrical refractive errors are also provided in **Table 1**.

**Table 1:** The demographic data of the study subjects including number of eyes, age, sphere, and cylinder.

	Total	Simple Myopia	Compound Myopic Astigmatism	P-value
Number of eyes	1370	648	722	–
Age (year)	29.01 ± 5.19	28.85 ± 5.05	29.10 ± 5.31	0.43
Spherical Equivalent (D)	-3.64 ± 1.59	-3.31 ± 1.37	-4.07 ± 1.64	P < 0.0001
Cylinder (D)	-1.01 ± 0.95	-0.32 ± 0.18	-1.49 ± 0.84	P < 0.0001
J0	0.26 ± 0.50	.03 ± 0.14	0.50 ± 0.60	P < 0.0001
J45	0.04 ± 0.30	0.01 ± 0.11	0.07 ± 0.40	0.009

A Spearman’s correlation test of independence was performed to determine if myopia will be independent of HOAs in the studied subjects (**Table 2**). Significant correlations were found between SEQ myopia and total RMS wave-front error (r = -0.10, P < 0.005).

**Table 2:** Spearman’s correlations between refractive error components and higher-order aberrations.

	Spherical equivalent (*r*)	J0 (*r*)	J45 (*r*)
Horizontal coma (Z_3_^1^)	-0.10	-0.18	-0.05
Vertical coma (Z_3_^-1^)	0.02	0.10†	0.004
Oblique trefoil (Z_3_^3^)	-0.06	-0.10†	0.10*
Vertical trefoil (Z_3_^-3^)	-0.007	-0.10*	-0.20†
Spherical aberration (Z_4_^0^)	-0.02	-0.03	0.05
Total root mean square wave-front error	-0.10†	0.05	0.10†

*Bonferroni corrected P <0.05, †Bonferroni corrected P <0.005.

Significant correlations were found between J0 and vertical coma-(Z_3_^-1^) (r = 0.10, P < 0.005); J0 and oblique trefoil (Z_3_^3^), (r = -0.10, P < 0.005); and J0 and vertical trefoil (Z_3_^-3^), (r = -0.1, P = 0.04). Increasing WTR astigmatism correlates positively with vertical coma (Z_3_^-1^) and negatively with oblique trefoil (Z_3_^3^) and vertical trefoil trefoil (Z_3_^-3^).

Significant correlations were found between J45 and oblique trefoil (Z_3_^3^), (r = 0.1, P = 0.02); J45 and vertical trefoil (Z_3_^-3^), (r = -0.20, P < 0.005); and J45 and total RMS wave-front error (r = 0.10, P < 0.005). (Provided P-values are after applying Bonferroni correction).

**Figure 1:**
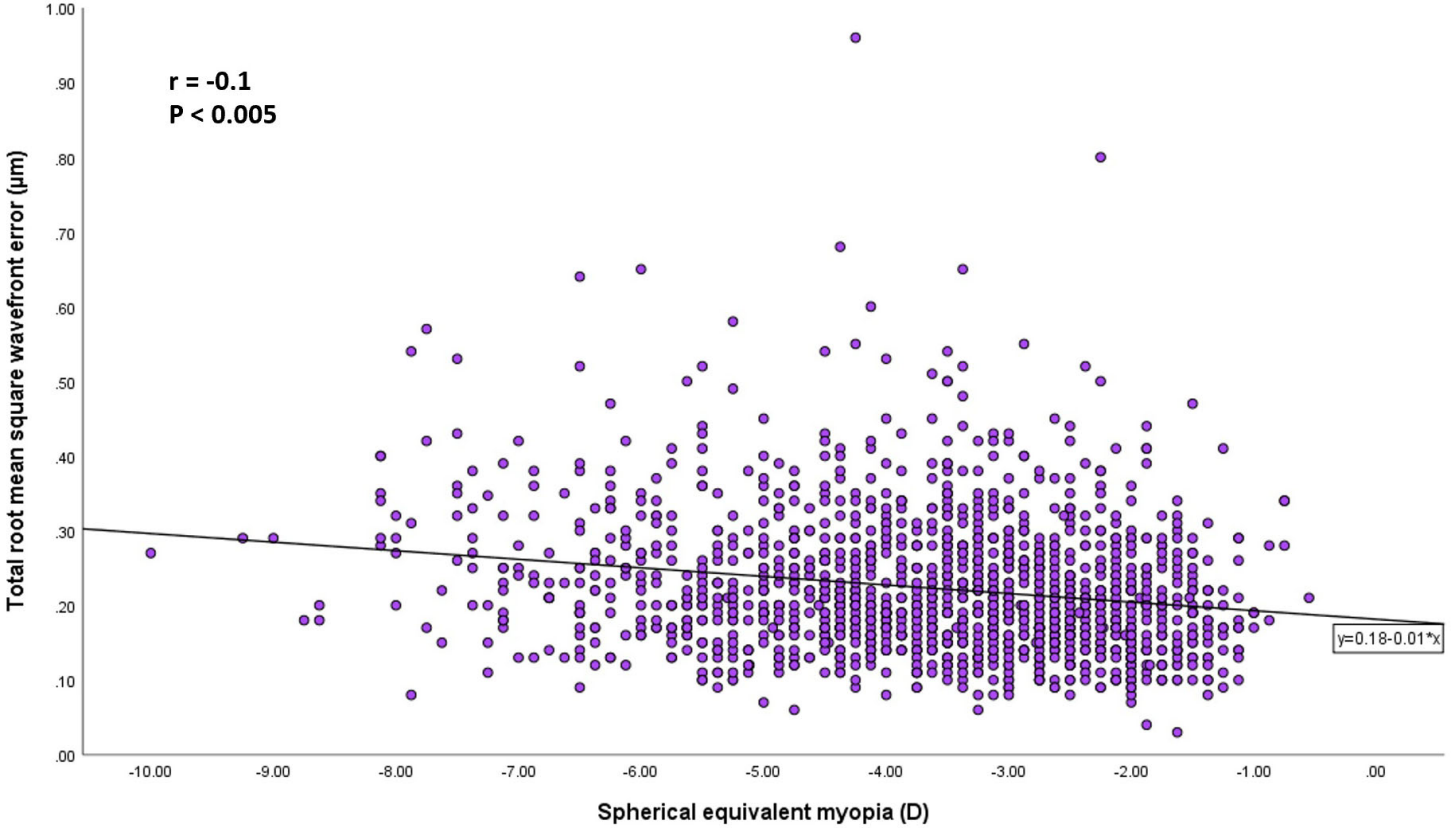
Scatterplots showing the spherical equivalent myopia vs the total root mean square wave-front error. There was a significant correlation between the parameters (Pearson correlation coefficient; r = −0.1, P < 0.005).

A multiple regression analysis was performed to find out the effect of HOAs on SEQ myopia controlling for age, gender, and corneal characteristics. The effect of total RMS wave-front error was significant (β = -0.10, P = 0.005). Among the controlled factors, the effects of age (β = 0.1, P = 0.004), mean keratometry front (β = -0.21, P < 0.001), and central corneal thickness (β = -0.10, P = 0.004) were found to be significant (**Figure 1**).

**Figure 2:**
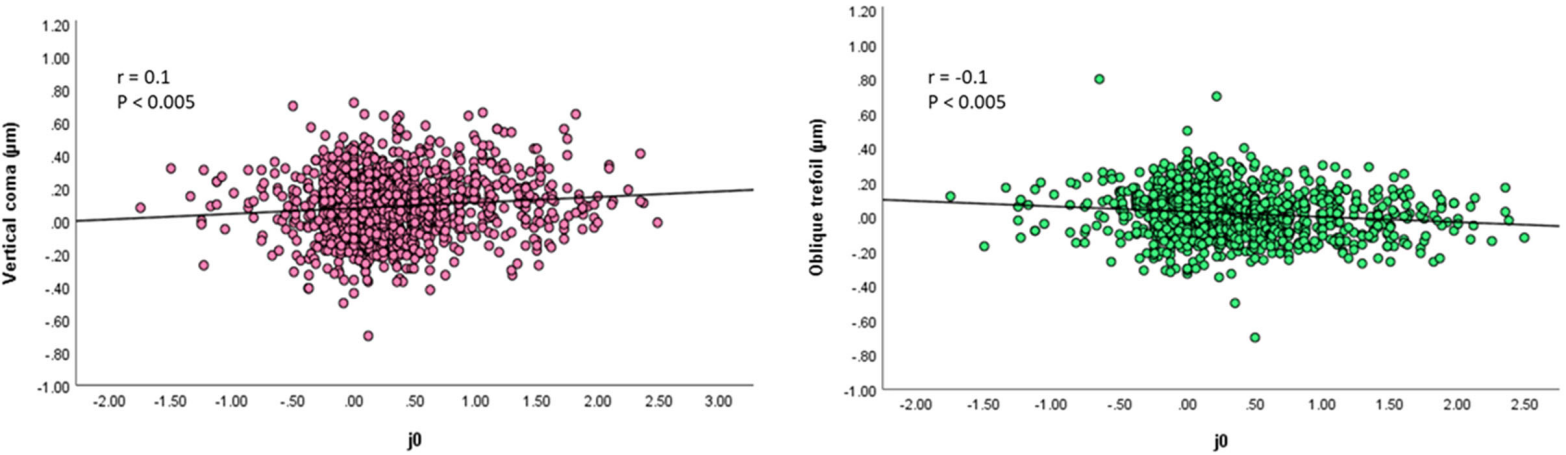
Right picture: Scatterplots showing the spherical J0 vs vertical coma. There was a significant correlation between the parameters (Pearson correlation coefficient; r = 0.1, P < 0.005). Left picture: Scatterplots showing the spherical J0 vs oblique trefoil. There was a significant correlation between the parameters (Pearson correlation coefficient; r = −0.1, P < 0.005).

A multiple regression analysis was performed to find out the effect of HOAs on J0 controlling for age, gender, and corneal characteristics. The effects of vertical coma (Z_3_^-1^) and oblique trefoil (Z_3_^3^) were significant (β = 0.10, P = 0.002 and β = -0.15, P < 0.001, respectively). Among the controlled factors, the effects of white-to-white corneal diameter (β = 0.17, P < 0.001) and anterior chamber depth (β = -0.11, P < 0.001) were found to be significant (**Figure 2**).

**Figure 3:**
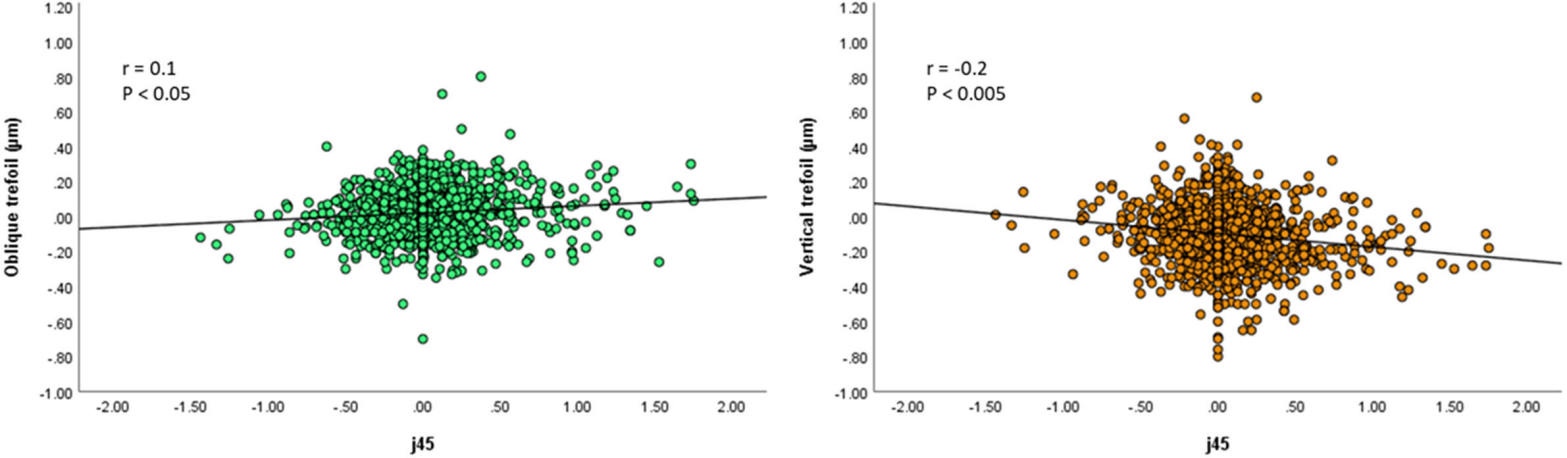
Right picture: Scatterplots showing the spherical J45 vs oblique trefoil. There was a significant correlation between the parameters (Pearson correlation coefficient; r = 0.1, P < 0.05). Left picture: Scatterplots showing the spherical J45 vs vertical trefoil. There was a significant correlation between the parameters (Pearson correlation coefficient; r = −0.2, P < 0.005).

A multiple regression analysis was performed to find out the effect of HOAs on J45 controlling for age, gender, and corneal characteristics. The effects of oblique trefoil (Z_3_^3^) and vertical trefoil (Z_3_^-3^) were significant (β = 0.10, P = 0.001 and β = -0.12, P < 0.001, respectively). None of the controlled factors was found to have significant effect on J45 (**Figure 3**).

### Comparison of HOAs as a function of SMY and CMA

**Figure 4:**
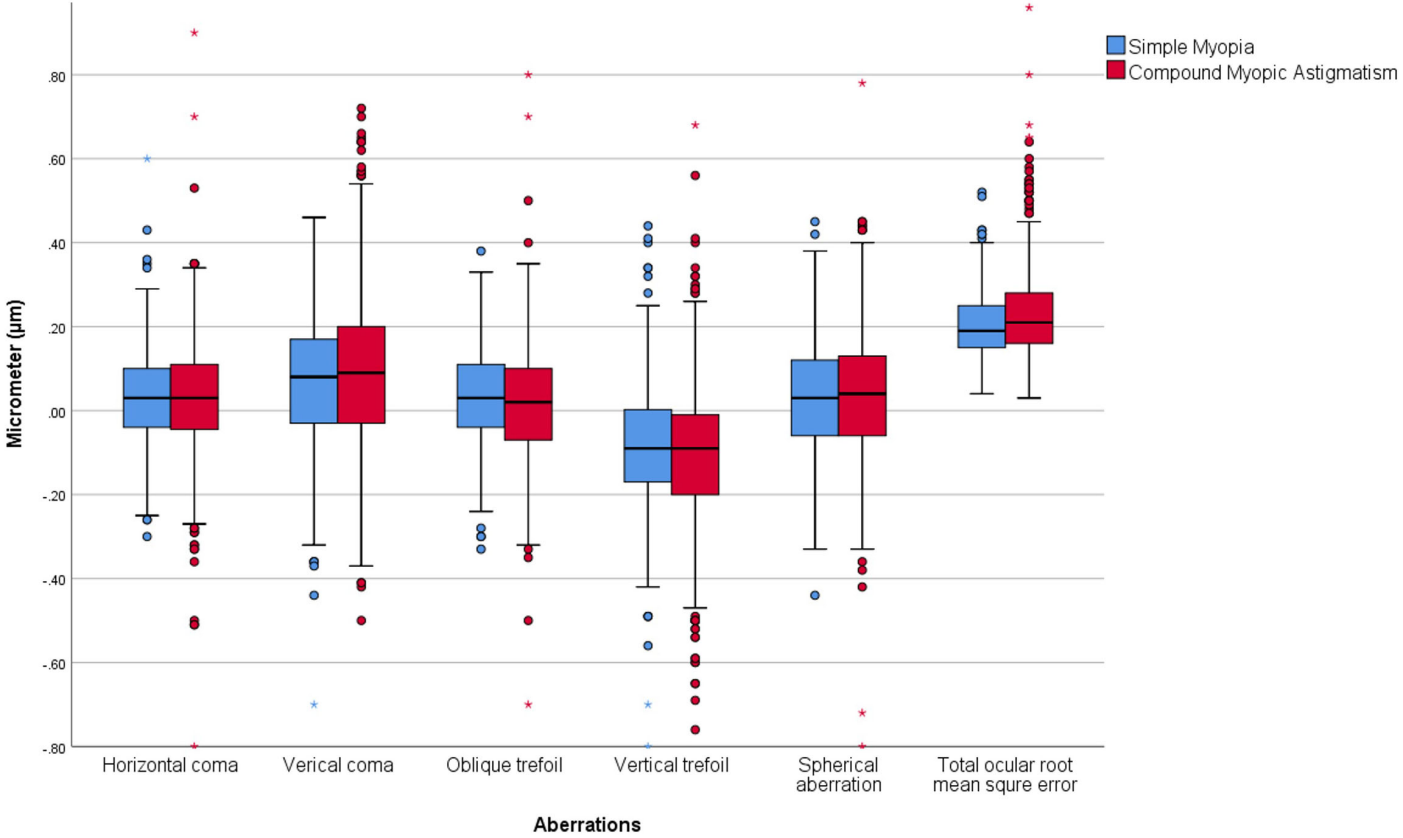
Showing box plots including error bars and range of the higher order aberrations for simple myopia and compound myopic astigmatism subgroups.

The comparisons of mean HOAs between CMY and CMA subgroups are provided in **Table 3**. The distributions of HOAs as box plots, including error bars and range of the HOAs according to types of refractive errors, are provided in **Figure 4**.

**Table 3:** Mean higher order aberrations in simple myopia, and compound myopic astigmatism subgroups.

	Total	Simple myopia	Compound myopic astigmatism	P-value
Horizontal coma (Z_3_^1^) (µm)	0.03 ± 0.12	0.03 ± 0.12	0.02 ± 0.13	0.50
Vertical coma (Z_3_^-1^) (µm)	0.09 ± 0.18	0.07± 0.17	0.10 ± .18	P < 0.001
Oblique trefoil (Z_3_^3^) (µm)	0.02 ± 0.13	0.03 ± 0.12	0.01 ± 0.13	0.04
Vertical trefoil (Z_3_^-3^) (µm)	-0.10 ± 0.16	-0.09 ± 0.16	-0.11 ± 0.15	0.02
Spherical aberration (Z_4_^0^) (µm)	0.03 ± 0.14	0.03 ± 0.14	0.03 ± 0.15	0.10
Total root mean square wave-front error	0.35 ± 0.17	0.34 ± 0.16	0.37 ± 0.18	P < 0.001

The mean values of total RMS wave-front error in SMY and CMA subgroups were 0.34 ± 0.16 µm and 0.37 ± 0.18 µm, respectively (P < 0.001). The SMY subgroup had a significantly smaller total RMS wave-front error value compared to the CMA subgroup.

The mean values of horizontal coma (Z_3_^1^) and spherical aberration (Z_4_^0^) in the SMY subgroup were 0.03 ± 0.12 µm and 0.03 ± 0.14 µm, respectively, and in the CMA subgroup, were 0.02 ± 0.13 µm and 0.03 ± 0.15 µm, respectively (P = 0.50, P = 0.10, respectively). The differences in the mean values of horizontal coma (Z_3_^1^) and spherical aberration (Z_4_^0^) were insignificant between the two subgroups.

The mean values of vertical coma (Z_3_^-1^), oblique trefoil (Z_3_^3^), and vertical trefoil (Z_3_^-3^) in the SMY subgroup were 0.07 ± 0.17 µm, 0.03 ± 0.12 µm, and -0.09 ± 0.16 µm, respectively, and in the CMA subgroup were 0.10 ± 0.18 µm, 0.01 ± 0.13 µm, and -0.11 ± 0.15 µm, respectively (P < 0.001, P = 0.04, P = 0.02, respectively). The absolute values of vertical coma (Z_3_^-1^) and trefoil were greater in the CMA subgroup compared to the SMY subgroup, and the absolute value of oblique trefoil (Z_3_^3^) was greater in the SMY subgroup compared to the CMA subgroup. The differences between the two subgroups are small and not clinically significant. However, studying the relationship between lower-order and HOAs, the statistically significant differences could be considerable.

## Discussion

Early investigations on the potential relationship between HOAs and myopia mostly showed a slight increment in the magnitude of HOAs with increasing myopia. However, systematic relationships showing a causative effect between components of HOAs and myopia were not reported (9, 18–22). However, recent longitudinal studies suggest some evidence of a relationship between HOAs and myopia or axial length elongation (6, 23, 24). Hiroka et al. represented that eyes with larger amounts of corneal HOAs showed less myopia progression and smaller axial length elongation, suggesting that corneal HOAs play a role in the refractive error and ocular developments in children (23).

As in previous cross-sectional studies (9, 20, 25, 26), in the current study, we found a slight increment in the magnitude of total RMS wave-front error with increasing SEQ myopia. The present study showed that total RMS wave-front error and the vertical third-order aberrations in CMA are higher than the SM subgroup, and this finding is in line with the results of previous studies (21, 27–29). Salman et al (28), found that myopic astigmatism individuals have the highest magnitude of trefoil and coma. Karimian et al. (25) found that vertical coma (Z_3_^-1^), vertical trefoil (Z_3_^-3^), and spherical aberration (Z_4_^0^) are predominant aberrations in CMA patients. It is important to note that the values of differences between the two subgroups are small, and such small differences do not interfere with individuals’ visual acuity in the CMA subgroup (30). Still, it shows the effect of astigmatism in this subgroup.

Interestingly, in the current study, we found a small but highly significant correlation between ocular third-order aberrations and the direction of astigmatism. The current study showed that increasing J0 (increasing WTR astigmatism) positively correlates with vertical coma (Z_3_^-1^) and negatively correlates with oblique trefoil (Z_3_^3^) and vertical trefoil (Z_3_^-3^). In line with our findings, Miller et al. (31), in a study on children subjects with a high prevalence of astigmatism, found a negative correlation between J0 and oblique trefoil (Z_3_^3^). Leung et al. (27) reported that asymmetry in the inferior–superior of the cornea along the principal vertical meridian was significantly associated with the vertical trefoil (Z_3_^-3^) and vertical coma (Z_3_^-1^) of the cornea. These findings suggest a systematic relationship between the direction of astigmatism and third-order aberrations.

In a previous investigation, Buehren et al. (8) discussed the interactions between the pattern of HOAs and lower-order aberrations through a computational modeling study. The researchers elaborated that in the context of blur-driven compensatory response in patients with a combination of 0.2-0.3 µm of positive-oblique trefoil (Z_3_^3^), negative-vertical coma (Z_3_^-1^), and negative-spherical aberration (Z_4_^0^), the best retinal image is obtainable with the presence of myopia and WTR astigmatism. In the current study, we found an association between ATR astigmatism and a positive shift in oblique trefoil (Z_3_^3^) and a negative shift in the vertical coma (Z_3_^-1^), which, combined with the negative spherical aberration (Z_4_^0^), was suggested to provide a cue for myopia and WTR astigmatism development. The correlation between the astigmatism direction and the direction of the third-order aberrations indicated in the current study warrants further investigation on the infants and toddlers population who are in the age of emmetropization and astigmatism development.

In addition to the relationship between vertical coma (Z_3_^-1^) and astigmatism, some association between vertical coma (Z_3_^-1^) and myopia has been reported. Sun et al. (32) in a study on anisometropic children showed that higher myopic eyes had lower corneal coma than those of the contralateral eyes. Hiroka et al. (23) found that myopia progression in eyes with a higher value of vertical coma (Z_3_^-1^) is slower. However, our study did not find a correlation between myopia and vertical third-order aberration. The interaction between astigmatism and vertical third-order aberrations may have masked the relationship between coma and myopia; therefore, further investigation in subjects with low astigmatism levels and corneal toricity may be conclusive.

Kwan et al. (14) reported that spherical aberration (Z_4_^0^) makes a negative shift with increasing myopia, while other cross-sectional studies did not report a relationship between spherical aberration (Z_4_^0^) and increasing myopia power (19, 27, 33, 34). In the current study, the spherical aberration (Z_4_^0^) was also steady with increasing myopia. Little et al. (33) found no relationship between spherical aberration (Z_4_^0^) and myopia power. Still, they found a relationship between a higher level of negative spherical aberration (Z_4_^0^) and longer axial length. On the other hand, Zhang et al. (35) found that with increasing myopia, the spherical aberration (Z_4_^0^) of the posterior cornea becomes less negative. From previous findings and the finding of our study, it can be inferred that in myopic eyes, changes in some of the optical components, such as the posterior cornea and axial length, are associated with spherical aberration (Z_4_^0^) changes, but increasing myopia power is not correlated with spherical aberration (Z_4_^0^) changes. there is a well-known compensatory effect between positive spherical aberration (Z_4_^0^) of the cornea and negative spherical aberration (Z_4_^0^) of the crystalline lens (36). It can be concluded that with increasing myopia, the compensatory mechanisms for spherical aberration (Z_4_^0^) remain relatively intact; therefore, a relationship between spherical aberration (Z_4_^0^) and myopia power is not attainable unless with measurement of axial length in longitudinal studies (6) or studying active accommodation state.

This study has some limitation. In this cross-sectional study, we have mentioned a systematic association between HOAs and astigmatism, which suggests a potential causal relationship. The causal relationships can be established through a longitudinal study. Therefore, to provide insights into how changes in HOAs and refractive errors develop over time a longitudinal study is required, especially on the infants and toddlers population who are in the age of astigmatism development. Additionally, this study did not account for environmental factors and lifestyle choices, such as time spent on near-work activities. Addressing these factors could provide a more comprehensive understanding of the relationship between HOAs and spherocylindrical components.

In conclusion, a slight increment in total ocular RMS wave-front error for 6 mm pupil with increasing myopia and astigmatism was observed. A weak systematic correlations between third-order aberrations and astigmatism direction were found. Increasing ATR is associated with a positive shift in oblique trefoil (Z_3_^3^) and a negative shift in vertical coma (Z_3_^-1^). Studying the interaction between lower-order and HOAs in infants and toddlers who are in the age of astigmatism development is suggested. A relationship between myopia and HOAs components were not observed in this study design.

## Data Availability

The raw data supporting the conclusions of this article will be made available by the authors, without undue reservation.
